# SalivaSTAT: Direct-PCR and Pooling of Saliva Samples Collected in Healthcare and Community Setting for SARS-CoV-2 Mass Surveillance

**DOI:** 10.3390/diagnostics11050904

**Published:** 2021-05-19

**Authors:** Nikhil S. Sahajpal, Ashis K. Mondal, Sudha Ananth, Allan Njau, Pankaj Ahluwalia, Gary Newnam, Adriana Lozoya-Colinas, Nicholas V. Hud, Vamsi Kota, Ted M. Ross, Michelle D. Reid, Sadanand Fulzele, Alka Chaubey, Madhuri Hegde, Amyn M. Rojiani, Ravindra Kolhe

**Affiliations:** 1 Department of Pathology, Medical College of Georgia, Augusta University, GA 30901, USA; nsahajpal@augusta.edu (N.S.S.); amondal@augusta.edu (A.K.M.); SANANTH@augusta.edu (S.A.); pahluwalia@augusta.edu (P.A.); achaubey@bionanogenomics.com (A.C.); AROJIANI@augusta.edu (A.M.R.); 2 Department of Pathology, Aga Khan University Hospital, Nairobi 30270-00100, Kenya; allan.njau@aku.edu; 3 School of Chemistry and Biochemistry, Georgia Institute of Technology, Atlanta, GA 30332, USA; gn27@mail.gatech.edu (G.N.); alc6@gatech.edu (A.L.-C.); nick.hud@chemistry.gatech.edu (N.V.H.); 4 Department of Medicine, Medical College of Georgia, Augusta University, GA 30901, USA; vkota@augusta.edu; 5 Center for Vaccines and Immunology, University of Georgia, GA 30602, USA; tedross@uga.edu; 6 Department of Pathology, Emory University, GA 30322, USA; michelle.reid@emory.edu; 7 Center for Healthy Aging, Medical College of Georgia, Augusta University, Augusta, GA 30901, USA; sfulzele@augusta.edu; 8 Bionano Genomics Inc., San Diego, CA 92121, USA; 9 Global Laboratory Services, PerkinElmer, Waltham, MA 02451, USA; Madhuri.Hegde@PERKINELMER.COM

**Keywords:** saliva, extraction-free, RT-PCR, pooling

## Abstract

Objectives: Limitations of widespread current COVID-19 diagnostic testing exist in both the pre-analytical and analytical stages. To alleviate these limitations, we developed a universal saliva processing protocol (SalivaSTAT) that would enable an extraction-free RT-PCR test using commercially available RT-PCR kits. Methods: We optimized saliva collection devices, heat-shock treatment, and homogenization. Saliva samples (879) previously tested using the FDA-EUA method were reevaluated with the optimized SalivaSTAT protocol using two widely available commercial RT-PCR kits. A five-sample pooling strategy was evaluated as per FDA guidelines. Results: Saliva collection (done without any media) showed performance comparable to that of the FDA-EUA method. The SalivaSTAT protocol was optimized by incubating saliva samples at 95 °C for 30-min and homogenization, followed by RT-PCR assay. The clinical sample evaluation of 630 saliva samples using the SalivaSTAT protocol with PerkinElmer (600-samples) and CDC (30-samples) RT-PCR assay achieved positive (PPA) and negative percent agreements (NPAs) of 95.0% and 100%, respectively. The LoD was established as ~60–180 copies/mL by absolute quantification. Furthermore, a five-sample-pooling evaluation using 250 saliva samples achieved a PPA and NPA of 92% and 100%, respectively. Conclusion: We have optimized an extraction-free RT-PCR assay for saliva samples that demonstrates comparable performance to FDA-EUA assay (Extraction and RT-PCR).

## 1. Introduction

The emergence of COVID-19 in the city of Wuhan, China in December 2019 has rapidly evolved into a pandemic. Since then, severe acute respiratory syndrome coronavirus 2 (SARS-CoV-2) has infected more than 40,997,453 individuals across the globe and has resulted in at least 1,127,637 COVID-19 related deaths (https://coronavirus.jhu.edu/map.html, last accessed 21 October 2020). The high transmission rate, along with the high percentage of asymptomatic infected individuals, have been identified as the major reason for the spread of the disease. Under these circumstances, diagnostic testing for COVID-19 remains the most rational approach for containing the virus, and is of unprecedented importance, because if infected individuals are detected early in the course of their infection, globally implemented strategies such as quarantine and contact tracing can be more effective [1,2].

The diagnostic testing for COVID-19 has relied heavily on nasopharyngeal (NPS) or oropharyngeal swab (OPS) samples collected in universal/viral transport medium (UTM/VTM), followed by RT-PCR based assays that target selected regions of the SARS-CoV-2 *nucleocapsid* (*N*), *envelop* (*E*), *spike* (*S*) and/or *open reading frame* (*ORF*) genes [3]. However, the massive global demand for testing has reached a crisis level, with clearly identifiable regional disparities. The emergence of a second upsurge in infections in countries previously showing a decline in cases highlights the need for a rapid, sensitive, cost-effective, and mass population testing methodology that can be implemented on a global scale [4,5]. The major limitations of the current COVID-19 diagnostic testing regimen lie at both the pre-analytical and analytical stages. The pre-analytical variables that influence the performance of the tests pertain primarily to sample type. Although NPS remains the gold standard sample type recommended for COVID-19 diagnostic testing, the collection of NPS samples poses challenges that include exposure risk to healthcare workers, and supply chain constraints pertaining to swabs, transport media, and personal protective equipment, with self-collection being difficult and yielding less sensitive results. Furthermore, several reports have highlighted the relatively poor sensitivity of NPS samples in early infection and longitudinal testing [6,7,8]. The analytical variables that determine the performance of the test are a combination of factors that include the efficiency of RNA extraction, RNA purification, and the sensitivity of the RT-PCR reaction. RNA extraction and purification have been identified as the major rate-limiting steps in the global testing protocol, leading to increased turnaround time. Additionally, the prolonged turnaround time for results, as well as the need for expensive kits, automated instrumentation, and trained personnel, have created additional economic and technological constraints.

**Figure 1:**
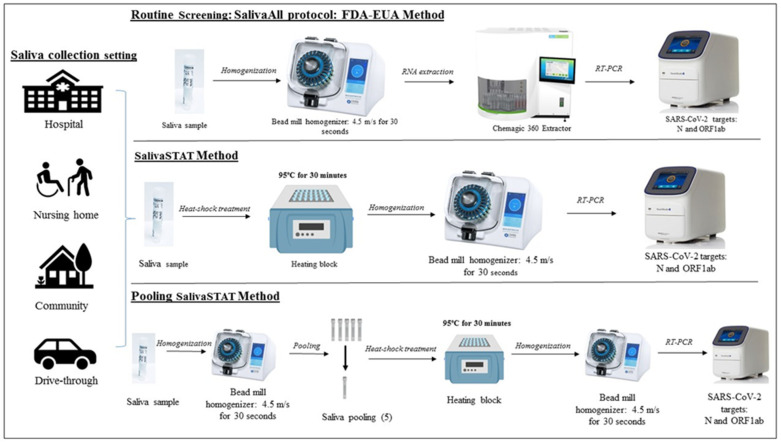
Schematic overview of sample processing and SARAS-CoV-2 assay workflow depicting main steps: Saliva samples collected in healthcare and community setting were tested and validated as follows: Upper panel: Saliva samples processed with SalivaAll protocol for nucleic acid extraction using a semi-automated instrument, followed by RT-PCR for N, ORF1ab gene targets and IC used as extraction and RT-PCR internal control; Middle panel: Saliva samples processed with SalivaSTAT method that included treatment of samples at 95 °C for 30 min and homogenization using a bead mill homogenizer followed by direct RT-PCR; Lower panel: Saliva samples homogenized using a bead mill before pooling samples with a five-sample pooling strategy followed by SalivaSTAT method for SARS-CoV-2 testing.

The scientific community has attempted to eliminate some of these pre-analytical and analytical constraints by utilizing saliva as a sample type and/or performing extraction-free RT-PCR assays, respectively. Several groups have shown comparable or higher sensitivity of saliva compared to NPS samples [9,10,11,12,13,14]. Although some conflicting reports have been published [15,16,17], we have previously optimized the processing of saliva samples and demonstrated higher sensitivity of saliva compared to NPS samples in both the healthcare and community setting [18]. Extraction-free RT-PCR assay eliminates the major limiting step in the analytic phase of COVID-19 testing. Several groups have demonstrated the feasibility of extraction free RT-PCR reaction maintaining the high sensitivity of the assay with NPS samples [19,20,21,22]. It is noteworthy that performing extraction free RT-PCR assay using saliva samples is a feasible method but only with the following caveats: (a) effective for the asymptomatic population; (b) requires early morning saliva (pure saliva); and, (c) has a limit of detection (LoD) of 6000–12,000 copies/mL [23]. Although the study is encouraging, the prerequisite conditions render it unsuitable for mass population screening, especially because the precise sample collection requirement and the low test sensitivity would lead to a high percentage of false-negative results. To address these limitations, we have developed and validated a highly sensitive (limit of detection ~60–180 copies/mL) extraction free RT-PCR assay (SalivaSTAT) using saliva samples collected in both the healthcare and community setting. The SalivaSTAT protocol enabled us to not only achieve high sensitivity but also simplified saliva processing, which allowed us to validate a five-sample pooling strategy using the SalivaSTAT- extraction free RT-PCR test (Figure 1).

## 2. Material and Methods

### 2.1. Study Site and Ethics

This single-center diagnostic study was conducted at Augusta University, GA, 30901, USA. This site is a CLIA accredited laboratory for high complexity testing and is one of the main SARS-CoV-2 testing centers in the State of Georgia, USA. The study was performed under AUIRB-HAC: 611298.

### 2.2. Patient Specimens and Setting

The study evaluated 879 saliva samples collected in either healthcare or community settings. Saliva samples were collected in 2 mL vials without any transport media. All samples were stored at 4 °C and transported to the SARS-CoV-2 testing facility at Augusta University within 12 h of collection. Of these 879 samples, 29 were used for assay optimization, 600 were used for clinical evaluation, and 250 were used for the pooling experiment.

### 2.3. Assay for the Detection of SARS-CoV-2 (FDA-EUA Method)

The assay is based on nucleic acid extraction followed by TaqMan-based RT-PCR assay to conduct in vitro transcription of SARS-CoV-2 RNA, DNA amplification, and fluorescence detection (PerkinElmer^®^ New Coronavirus Nucleic Acid Detection kit, FDA-EUA assay (PerkinElmer Inc., Waltham, MA, USA). The assay targets specific genomic regions of SARS-CoV-2: *nucleocapsid* (*N*) and *ORF1ab* gene. The TaqMan probes for the two amplicons are labeled with FAM and ROX fluorescent dyes, respectively, to generate target-specific signals. The assay includes an RNA internal control (IC, bacteriophage MS2) to monitor the processes from nucleic acid extraction to fluorescence detection. The IC probe is labeled with VIC fluorescent dye to differentiate its fluorescent signal from SARS-CoV-2 targets.

### 2.4. Routine Diagnostic Screening (RNA Extraction and RT-PCR)

All 879 saliva samples were tested using the FDA-EUA approved assay. In brief, the saliva samples were collected in 2 mL Omni tubes (2 mL reinforced tubes, SKU: 19-628D, Omni International, USA) and homogenized at 4.5 m/s for 30 s using the Omni bead mill homogenizer (Bead Ruptor Elite, SKU: 19-040E, Omni International, USA). An aliquot of 300 μL from each sample, positive and negative controls, was then added to respective wells in a 96 well plate. A 5 μL internal control (IC), 4 μL Poly(A) RNA, 10 μL proteinase K and 300 μL lysis buffer were then added to each well. The plate was placed on a semi-automated instrument (Chemagic 360 Instrument, PerkinElmer Inc.) following the manufacturer’s protocol. The nucleic acid was extracted in a 96 well plate, with an elution volume of 60 μL. From the extraction plate, 10 μL of extracted nucleic acid and 5μL of PCR master mix (3.75 μL reagent A, 0.75 μL reagent B, and 0.5 μL enzyme) were added to the respective wells in a 96 well PCR plate. The PCR method was set up as per the manufacturer’s protocol on Quantstudio 3 or 5 (ThermoFisher Scientific, Waltham, MA, USA). The samples were resulted as positive or negative, based on the Ct values specified by the manufacturer (Supplementary file 1).

### 2.5. Extraction-Free RT-PCR Assay (SalivaSTAT) Optimization

The following parameters were optimized: (a) Saliva collection devices, (b) Heat-shock treatment and homogenization; (c) Heat shock with and without homogenization; (d) Saliva sample homogenization.

### 2.6. Saliva Collection Devices

Saliva samples were collected in three different collection devices viz. DNA/RNA shield from Zymo Research (DNA/RNA Shield Saliva Sputum Collection Kit—DX, Cat. No R1210-E, Zymo Research, Irvine, CA, USA), Spectrum DNA from Spectrum solutions (cat no. SDNA-1000), and Omni tubes (2 mL reinforced tubes, SKU: 19-628D, Omni International, USA). The Zymo and Spectrum devices contain transport media that is mixed in a 1:1 ratio with saliva, whereas saliva collected in the Omni tubes was media-free. Four previously characterized SARS-Co-V-2 positive samples collected in each device were subjected to 95 °C for 10, 20, and 30 min respectively, followed by homogenization at 4.5 m/s for 30 s using the Omni bead mill homogenizer (Bead Ruptor Elite, SKU: 19-040E, Omni International, USA). Following homogenization, the samples were directly processed for RT-PCR using the PerkinElmer RT-PCR kit. The RT-PCR reaction was set up with 20 µL saliva sample and 10 µL reaction master mix (5.5 µL reagent A, 2 µL IC, 1.5 µL reagent B, 1 µL enzyme). The PCR method was set up as per the manufacturer’s protocol on Quantstudio 3 or 5 (ThermoFisher Scientific, USA). The samples were resulted as positive or negative, based on the Ct values specified by the manufacturer.

### 2.7. Heat-Shock Treatment and Homogenization

Our group and others have previously attempted to optimize the temperature required for direct RT-PCR for NPS samples [19,24]. The next step was to optimize the duration of heat treatment by subjecting four previously characterized positive saliva samples (used in Section 2.6) to 95 °C for 10, 20, and 30 min, respectively, followed by homogenization at 4.5 m/s for 30 s using the Omni bead mill homogenizer (Omni International, USA). Subsequently, the samples were directly processed for RT-PCR.

### 2.8. Heat Shock with and without Homogenization

Twenty-five saliva samples were subjected to 95 °C for 30 min, and an aliquot from each sample was either vortexed for 30 s or homogenized at 4.5 m/s for 30 s using the Omni bead mill homogenizer (Omni International, USA). Following the respective treatment, all samples were directly tested by RT-PCR.

### 2.9. Saliva Sample Homogenization

Our group has previously demonstrated the need for homogenization of saliva samples for optimized processing for SARS-Co-V-2 testing. Herein, we perform additional studies to demonstrate that optimal results are achieved using saliva samples for extraction-free RT-PCR using homogenization [18].

#### 2.9.1. Determination of Saliva Viscosity

Disposable viscometers were constructed from plastic tubing and plastic transfer pipettes, called Setup A and Setup B, respectively (Supplementary file 2, Figure S1). Standard curves for these viscometers were constructed using water-glycerol solutions and data reported by Segur and Oberstar [25] for the viscosity of water-glycerol mixtures at room temperature ranging from 1 cP (100% water, 0% glycerol) to 1400 cP (0% water, 100% glycerol). Several water-glycerol standards were loaded onto viscometer Setup A for low viscosity liquids (1 cP to 10 cP) and Setup B for higher viscous liquids (100 cP to 1400 cP). To generate the standard curves, the amount of time required for a specific weight of solvent to flow between two marks on each viscometer was plotted versus the reported viscosity of several water-glycerol mixtures. For Setup A, the viscometer was constructed from plastic tubing with an inner diameter of 1.19 mm and timing marks separated by 240 mm (Figure S1A). For Setup B, the viscometer was constructed from a wide-bore pipette (Cat # 13-711-6M, Fisher Scientific, Pittsburgh, PA, USA), with the top removed for easy loading, and timing marks separated by 50 mm (Figure S1B). Standard curves for both viscometers revealed excellent linear correlations between the measured time for water-glycerol samples to travel between timing marks and the reported viscosities of each mixture (Figure S2). The viscosities of saliva samples were measured by loading on either viscometer Setup A or Setup B and measuring the time required for travel between timing marks. The travel times were converted to viscosity measurements by using the standard curves shown in Figure S2. We note that our method for viscosity measurement represents an inexpensive and safe (disposable apparatus) adaptation of the Ostwald viscometer [26] which is based on Poiseuille’s law or Poiseuille’s equation. Briefly, Poiseuille’s equation for viscosity determination can be approximated to η = Aρt, where η corresponds to the viscosity, A is a constant associated to the viscometer, ρ is the density of the liquid and t is the time the liquid requires to travel a set distance for a given volume of the liquid at a particular temperature. If the Poiseuille equation applies to a solvent, then a plot of η/ρ versus travel time will reveal in a linear relationship. The excellent linear correlation of these values for the glycerol-water system confirms the proper functioning of our disposable viscometers (Figure S1).

#### 2.9.2. Weight Distribution

The entire saliva sample was transferred to a preweighed 1.5 mL Eppendorf tube and then weighed again to determine the total weight of the saliva. Samples were centrifuged at 4500× *g* for 2 min to pellet their high viscosity elements. The low viscous fraction was transferred back to the original tube and the remaining saliva was reweighed to determine the weight of the high viscosity fraction. The difference between the total weight and the weight of the high viscosity fraction provided the weight used for viscosity measurements.

### 2.10. SalivaSTAT: Clinical Sample Evaluation Using Commercial Kits

Six hundred previously tested saliva samples were evaluated using SalivaSTAT protocol and tested with modified Perkin Elmer Inc. (FDA-EUA) RT-PCR assay. The SalivaSTAT assay was optimized with the following conditions: saliva collected in the media-free Omni tubes was subjected to 95 °C for 30 min followed by homogenization at 4.5 m/s for 30 s using the Omni bead mill homogenizer (Omni International, USA). Following homogenization, the samples were directly tested with an RT-PCR assay using 10 µL of master mix (5.5 μL reagent A, 2 μL IC 1.5 μL reagent B, and 1 μL enzyme) and 20 µL of sample, and 30 saliva samples were repeated with the CDC RT-PCR assay.

### 2.11. Pooling Saliva Samples for Mass Population Screening

A five-sample pooling strategy was evaluated as per FDA guidelines (https://www.fda.gov/medical-devices/coronavirus-disease-2019-covid-19-emergency-use-authorizations-medical-devices/vitro-diagnostics-euas, last accessed 15 April 2021). Briefly, 25 previously confirmed positive saliva samples were identified to create 25 positive pools each comprised of one positive and four negative samples. For pooling, samples were homogenized (4.5 m/s for 30 s using the Omni bead mill homogenizer) and 50 µL of each sample was pooled (five samples in one vial) and processed as per SalivaSTAT protocol. The Ct values of positive samples ranged from (*N*: 19.8–36.8, *ORF1ab*: 25.3–Undetermined). Similarly, 25 negative sample pools were created comprised of five negative samples. All saliva samples were processed with the SalivaSTAT protocol and tested using the PerkinElmer RT-PCR assay.

### 2.12. Data Analysis

Data were analyzed for descriptive statistics and presented as a number (%) for categorical variables and mean ± standard deviation (SD) for continuous variables. Ct values were compared using Paired *t*-test. Regression analysis with slope and intercept along with a 95% confidence interval was determined in the pooling sample study.

## 3. Results

### 3.1. Saliva Collection Devices

For the saliva samples collected in Zymo, Spectrum, and Omni devices, the amplification for IC and SARS-CoV-2 *N* and *ORF1ab* target genes was observed only in saliva samples collected in Omni vials (which were devoid of any media), whereas no amplification was observed in saliva samples collected in Spectrum or Zymo devices. Thus, the process variables for extraction free PCR were optimized using saliva samples collected in Omni devices.

### 3.2. Heat-Shock Treatment and Homogenization

**Figure 2:**
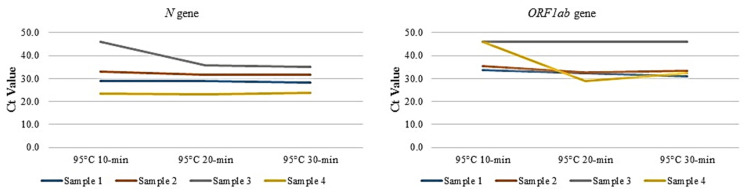
*N* and *ORF1ab* gene Ct values of four previously tested positive samples treated at 95 °C for 10, 20, and 30 min followed by homogenization and direct RT-PCR. Ct value of 45 was considered as undetermined and used for plotting.

Four previously tested positive samples were subjected to 95 °C for 10, 20, and 30 min followed by homogenization and direct RT-PCR. Of the four samples, the Ct values for *N* and *ORF1ab* gene were comparable at all three conditions. However, in samples 3 and 4, the Ct value for the *ORF1ab* gene, and in sample 3, the Ct value of the N gene, remained undetermined at 10 min treatment, whereas it was comparable at 20 and 30 min treatment (Figure 2).

### 3.3. Heat-Shock Treatment with and without Homogenization

**Figure 3:**
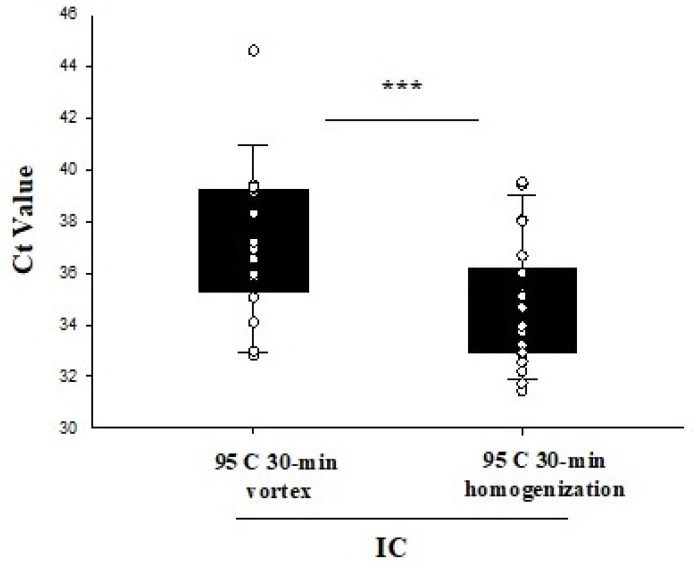
IC Ct values of twenty-two saliva samples subjected to 95 °C for 30 min, and either vortexed for 30 s or homogenized followed by direct RT-PCR. *** *p* < 0.001.

Twenty-five saliva samples (25 negative and 1 positive) were subjected to 95 °C for 30 min, and an aliquot from each sample was either vortexed for 30 s or homogenized followed by direct RT-PCR. The Ct values for IC (37.12 ± 2.93 vs. 35.03 ± 2.36) were significantly higher in samples that were vortexed compared to homogenization. The amplification curve for the positive sample did not result in an S-shaped curve with vortexing, while a standard amplification curve with comparable Ct value to the FDA-EUA method was identified with the homogenization method. Furthermore, six samples remained invalid with the vortex protocol compared to no invalid samples with the homogenization method (Figure 3).

### 3.4. Saliva Sample Homogenization

The effect of homogenization of the saliva samples was evaluated by performing viscosity measurement studies.

#### 3.4.1. Viscosity Determination

The various saliva samples were loaded onto either of two types of readily-fashioned, disposable viscometers (see Methods for more information) to obtain the time required for each sample to pass between two timing markings (see Supplemental Table S1A,B). The average time for each sample was divided by the sample density and these values were compared to the standardized curves Figure S2A,B, to determine the sample viscosity, as shown in Supplemental Table S2. The unprocessed samples had the highest viscosity ranging from 176 cP to 677 cP (between the viscosity of olive oil and honey), compared to processed samples with 2.1 cP to 3.1 cP, which have a viscosity close to the viscosity of water (1 cP).

#### 3.4.2. Weight Distribution

Saliva samples do not have uniform consistency, and vary from watery, thick, sticky, to frothy depending on the amount of proteins present [27]. For viscosity measurements, it was necessary to use a benchtop centrifuge to separate nonflowable material from the flowable material that could be run through a viscometer. To determine the percentage of flowable material that was used for the viscosity studies, it was necessary to separate and weigh these two phases of the saliva material (see Methods for more information). The inconsistency of the unprocessed samples spanned a range of 61.2% to 98.4%, and nonflowable material that could not be used in viscosity measurements (Table S3).

### 3.5. SalivaSTAT: Clinical Sample Evaluation Using Commercial Kits

**Figure 4:**
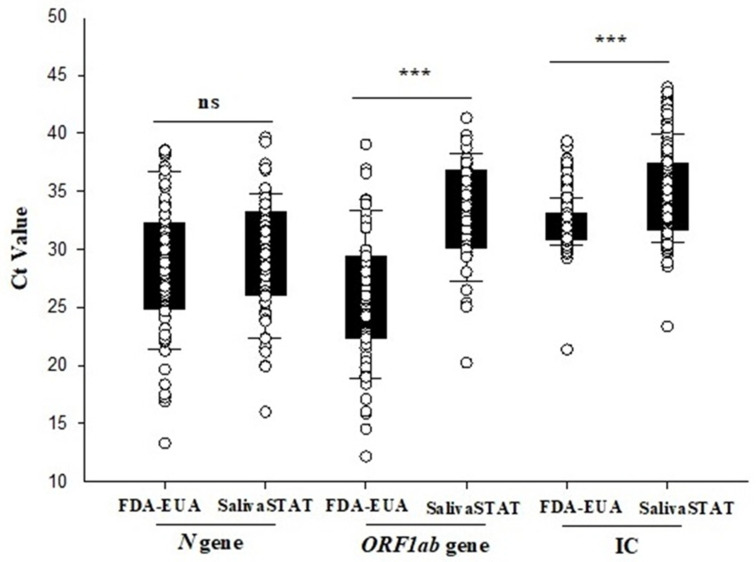
Comparison of Ct values for *N*, *ORF1ab* genes, and IC of 600 saliva samples evaluated with FDA-EUA (RNA extraction and RT-PCR) method and SalivaSTAT (Direct RT-PCR) method. *** *p* < 0.001.

The SalivaSTAT method was optimized with the following conditions: saliva collected in the media-free Omni tubes was subjected to 95 °C for 30 min followed by homogenization at 4.5 m/s for 30 s using the Omni bead mill homogenizer (Omni International, USA). Following homogenization, the samples were directly tested with an RT-PCR assay. Six hundred (600) saliva samples, comprised of 61 positive and 539 negative samples, were tested with the SalivaSTAT method using the PerkinElmer RT-PCR assay. The Ct values (mean ± SD) for the *N* gene were comparable (29.3 ± 4.8 vs. 28.3 ± 5.6), whereas the Ct value for IC (34.5 ± 3.7 vs. 32.2 ± 1.9) and *ORF1ab* (33.0 ± 4.3 vs. 25.9 ± 5.5) genes were significantly higher with SalivaSTAT compared to FDA-EUA method, respectively (Figure 4).

**Figure 5:**
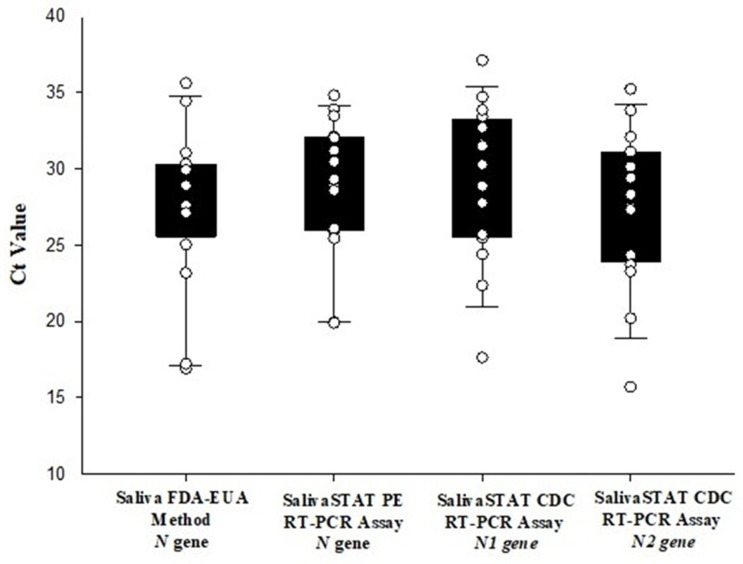
Comparison of *N* gene Ct values of saliva samples evaluated with FDA-EUA method, and SalivaSTAT method using PerkinElmer Inc. (PE) RT-PCR assay and CDC RT-PCR assay.

Of the 61 positive samples, 95.0% (58/61) were accurately detected by the SalivaSTAT method compared to the FDA-EUA method. The positive samples were selected to represent both strong and weak positives, with Ct values ranging from 16.8–38.5 for the *N* gene, and 14.5–39.0 for the *ORF1ab* gene with the FDA-EUA method. Three very weak positive samples (with Ct values of *N*: 38.5, 38.4, 38.2; *ORF1ab*, Und., 36.9, Und., respectively) identified with the FDA-EUA method were not detected with the SalivaSTAT method. Of the 539 negative samples, 509 resulted as negative and 30 as invalid. It must be noted that 5% (30/600) samples resulted as invalid with the SalivaSTAt method. Similarly, 30 saliva samples, comprised of 16 positive and 14 negative samples were tested with the SalivaSTAT method using the CDC RT-PCR assay. The Ct values of the 16 positive samples for *N* gene [27.6 ± 5.1 vs. 28.8 ± 4.4 vs. *N1*: 27.5 ± 5.1, *N2*: 29.1 ± 5.0) were found to be comparable with the FDA-EUA method and SalivaSTAT-PerkinElmer RT-PCR assay, respectively (Figure 5). The overall positive and negative percent agreement was found to be 96% and 100%, respectively. The LOD was determined to be ~60–180 copies/mL by absolute quantification calculation.

### 3.6. Pooling Saliva Samples for Mass Population Screening

**Figure 6:**
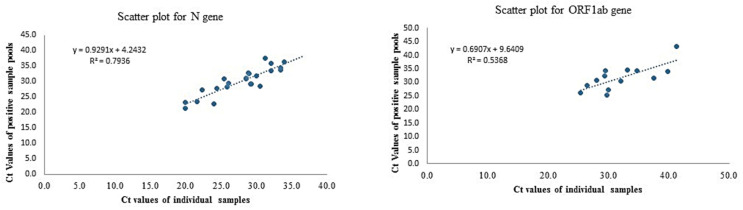
The Ct values comparison of *N* and *ORF1ab* gene with individual testing vs. pool testing.

The five-sample pooling strategy was evaluated by comparing the results of the 25 positive and negative pools to individual sample testing results. The pooled testing results demonstrated a 92% positive and 100% negative percent agreement. The *N* and *ORF1ab* gene Ct values were compared between pooled and individual testing. Regression analysis with slope and intercept along with a 95% confidence interval was determined. The shift in Ct value was found to be significant with pooled testing towards higher Ct values, nonetheless, the pools containing positive samples with viral loads close to the assay’s LoD (i.e., weak positives) were accurately detected (Figure 6).

## 4. Discussion

The COVID-19 pandemic has placed an enormous burden on the health care systems globally, to the point of exhausting currently available resources to manage and/or contain its spread. This has led groups to explore alternative methods to diagnose COVID-19 [28]. However, testing for SARS-CoV-2 has been the most critical measure implemented across the globe [1]. The current COVD-19 diagnostic testing regimen primarily relies on NPS samples, followed by qualitative RT-PCR-based methods for the detection of SARS-CoV-2. However, several limitations exist in the current methodology at both pre-analytical and analytical stages. In the pre-analytical stage, NPS is associated with exposure risk to healthcare workers, high cost, invasive collection, and supply-chain constraints [6,7,8]. Furthermore, the RNA extraction step in the analytical stage is the most significant rate-limiting step in this protocol because of a wide range of reasons that include, the requirement for competent testing personnel, cost of reagents/kits, equipment, and turnaround time.

To overcome the pre-analytical and analytical limitations of current COVID-19 testing methods, saliva samples and extraction-free RT-PCR assays have recently been explored. Significant efforts have been made to develop an extraction-free RT-PCR assay using NPS swabs, and several groups have optimized dry swabs, transport media, heat inactivation, different RT-PCR reaction chemistries, and RT-PCR methods [19,20,21,22], but minimal information has emerged on extraction-free RT-PCR assay using saliva samples. To our knowledge, only one report has evaluated the performance of extraction-free RT-PCR assay using saliva samples, and this assay was limited to an asymptomatic population, used early morning saliva, and yielded low sensitivity [23]. Hence, the goal of this study was to optimize both pre-analytical and analytical variables by developing a universal saliva processing protocol that would enable an extraction-free RT-PCR test using any of the commercially available RT-PCR kits.

In the pre-analytical stage, the most important variables are the collection method and the collection device. Several studies have demonstrated comparable or higher sensitivity of early morning saliva, deep throat saliva, and typical saliva compared to NPS samples, in both the healthcare and community settings [9,10,11,12,13,14]. Furthermore, most of these studies have used specialized saliva collection devices that mix saliva in a 1:1 ratio with a transport media. In the present investigation, saliva samples collected in two specialized collection devices (with media), and one in-house collection device (without media) were evaluated for extraction-free RT-PCR assay. The saliva samples collected in specialized collection devices did not show amplification for either internal control or the two SARS-CoV-2 *N* and *ORF1ab* gene targets, whereas the saliva samples collected in the in-house collection devices showed amplification for each target with Ct values comparable to the FDA-EUA method. This is consistent with our previous report on extraction-free RT-PCR assay using NPS samples, where NPS samples collected in VTM/UTM did not show amplification for any of the three targets.

The VTM/ UTM appears to inhibit the PCR reaction and is a consistent observation, as several groups developing the extraction-free RT-PCR assay have either designed their PCR chemistries or have validated the input of sample that would allow amplification in their respective RT-PCR methods [19,22]. In addition to being cost-prohibitive and difficult to implement globally, designing alternate PCR chemistries would be challenging in achieving high sensitivity. We, therefore, attempted to collect saliva samples in the in-house collection device (media-free) which is in alignment with a previous report [23].

We and others have also previously optimized the temperature required for direct RT-PCR for NPS samples [19,24], and thus, our aim was to optimize the duration of the temperature treatment by subjecting four previously characterized positive saliva samples to 95 °C for 10, 20, and 30 min followed by homogenization. Of the four samples, the Ct values for IC, *N,* and *ORF1ab* gene were compared with all three conditions. However, in samples three and four, the Ct value for *N* and *ORF1ab* gene remained undetermined at 10 min intervals, whereas it was comparable at 20 and 30-min intervals, respectively. Thus, a 30-min incubation time was deemed optimal for further experiments, as, in addition to comparable Ct value results, the 30-min interval would inactivate the virus rendering it safe to process in clinical and nonclinical laboratories around the globe. The importance of homogenization of saliva samples after the 30-min incubation at 95 °C, was evident from the significantly lower Ct values for IC, *N*, and *ORF1ab* targets compared to the samples subjected to vortexing alone.

Homogenization also addresses several critical issues associated with saliva samples. Saliva samples collected in specialized devices or without the use of media are difficult to pipet by testing personnel, which leads to increased processing time [29]. In addition, the gel-like consistency of saliva samples has led to lower sensitivity and resulted in a higher percentage of invalid results. Saliva samples do not have uniform consistency, varying from watery to thick, sticky, or frothy, depending on the amount of constituent proteins. We have previously demonstrated the importance of homogenization of saliva samples, which not only eliminates the processing challenges but also renders them more sensitive compared to NPS samples [18]. We also evaluated the viscosity of saliva samples before and after homogenization. The unprocessed samples had the highest viscosity ranging from 176 cP to 677 cP compared to the processed samples with 2.1 cP to 3.1 cP, which have a viscosity close to that of water (1 cP). This observation highlights and explains the difficulty these unprocessed samples would pose inaccurate pipetting and during the extraction procedure, where uniform mixing of reagents would be challenging. Thus, to eliminate processing challenges and taking cues from our previously published studies that demonstrate the role of homogenization in increasing the sensitivity of saliva samples, we processed each sample with the homogenization step.

The 600-sample clinical evaluation of this optimized extraction-free RT-PCR assay (SalivaSTAT protocol) using two commercial kits, demonstrated an overall positive and negative percent agreement of 96% and 100%, respectively. Interestingly, the Ct value for the SARS-CoV-2 *N* gene with the SalivaSTAT protocol was comparable to that of the FDA-EUA method. The Ct value for *ORF1ab* and IC were significantly higher with SalivaSTAT compared to the FDA-EUA method. These results are in alignment with previously published reports on heat-inactivated direct PCR assay using NPS samples, where comparable Ct values were observed for the N1 gene compared to other targets (E and ORF). Heat treatment cleaves the RNA into short fragments and the best results are obtained with the N1 gene primers, as reported previously [19]. Only three samples that were very weakly positive were not detected with the SalivaSTAT method. It is recommended that samples be given after rinsing the mouth with water and fasting for 2 h, as residual food/beverages, medications, recreational products such as cigarette smoke residues, and oral hygiene products such as toothpaste and gargles can inhibit the RT-PCR reaction, given that no RNA extraction/purification step is involved in the extraction-free protocol. As the instructions are not always followed, it may have accounted for the invalid results with the SalivaSTAT method using the PerkinElmer RT-PCR. The evaluation using the CDC RT-PCR kit might be deemed more suitable for extraction-free assays, because the assay employs an N gene target, and the housekeeping *RnaseP* gene target is extracted in abundance which would lead to zero or minimal invalid results. The salivaSTAT is cost-effective method, as it does not require RNA extraction kits or automatic extractors and 96 samples can be reported in ~3 h (Table 1).

In addition to clinical sample evaluation with the SalivaSTAT protocol, we were also able to successfully validate saliva samples with a five-sample pooling strategy. The pooled testing results demonstrated a positive percent agreement of 92% (23/25 pools showing positive results), with two pools that contained the sample with a very high Ct value being undetectable. The negative percent agreement was found to be 100%. We have previously demonstrated the feasibility and accuracy of a sample pooling approach with NPS and saliva samples for wide-scale population screening for COVID-19. Herein, we extend the utility and potential benefits of the sample pooling approach for population screening using the SalivaSTAT protocol for saliva samples.

Considering the evolving epidemiology of COVID-19 and the reopening of educational and professional institutions, travel, tourism, and social activities, monitoring SARS-CoV-2 will remain a critical public health need for the near future. Therefore, the use of a noninvasive diagnostic test (i.e., saliva collection) and extraction-free RT-PCR methodology will significantly enhance screening and surveillance activities. Taken together, we have optimized an extraction-free direct RT-PCR assay for saliva samples that demonstrated comparable performance to FDA-EUA assay (extraction and RT-PCR). The SalivaSTAT protocol is a rapid, sensitive, and cost-effective method that can be adopted globally, has the potential to accelerate testing needs, and could play a significant role in helping to curb the current pandemic.

## Figures and Tables

**Table 1:** Comparison between Routine diagnostic assay and SalivaSTAT method.

	Routine Diagnostic Assay	SalivaSTAT
Saliva collection devices	Specialized devices containing VTM/UTM or Saliva collected without media.	Saliva collected without media
Homogenizer	Required	Required
RNA extraction	Manual or automated	Not required
RT-PCR	Required	Required
Limit of Detection	20–60 copies/mL	60–180 copies/mL
TAT (96 samples)	~5 h	~3 h

## Data Availability

All relevant data is made available in the manuscript and supplementary files.

## Supplementary Materials

The following are available online at https://www.mdpi.com/article/10.3390/diagnostics11050904/s1. Supplementary file 1. Result interpretation based on Ct cutoff values as per manufacturer’s recommendations. Supplementary file 2. Figure S1: Home-built, disposable viscometers. Figure S2: Standard curves for viscosity determination. Supplemental Table S1: Raw data of saliva samples. Supplemental Table S2: Viscosity of saliva samples. Supplemental Table S3: Weight Distribution.
