# Supplementary material for: SalivaSTAT: Direct-PCR and Pooling of Saliva Samples Collected in Healthcare and Community Setting for SARS-CoV-2 Mass Surveillance
Source: Diagnostics (Basel). 2021 May 19;11(5):904. doi: 10.3390/diagnostics11050904 (PMC8159081; doi:10.3390/diagnostics11050904)
Supplement: Supplementary file 1 [file diagnostics-11-00904-s001.zip › diagnostics-1211909-supplementary.pdf]

Supplementary file 1

Supplement file 1. Result interpretation based on Ct cutoff values as per manufacturer's recommendations.

Ct		Result Interpretation
IC (VIC/HEX)	N(FAM), ORF1ab (ROX)	
≤ 40	Both target undetermined or >42	SARS-Cov-2 not detected
\	Both targets ≤ 42	SARS-Cov-2 detected
\	One of the targets ≤ 42	SARS-Cov-2 detected
>40 or undetermined	Both target undetermined or >42	Invalid result

Supplementary file 2

Supplementary information for "SalivaSTAT: Direct-PCR and pooling of saliva collected in healthcare and community setting for SARS-CoV-2 mass surveillance"

Setup A Setup B

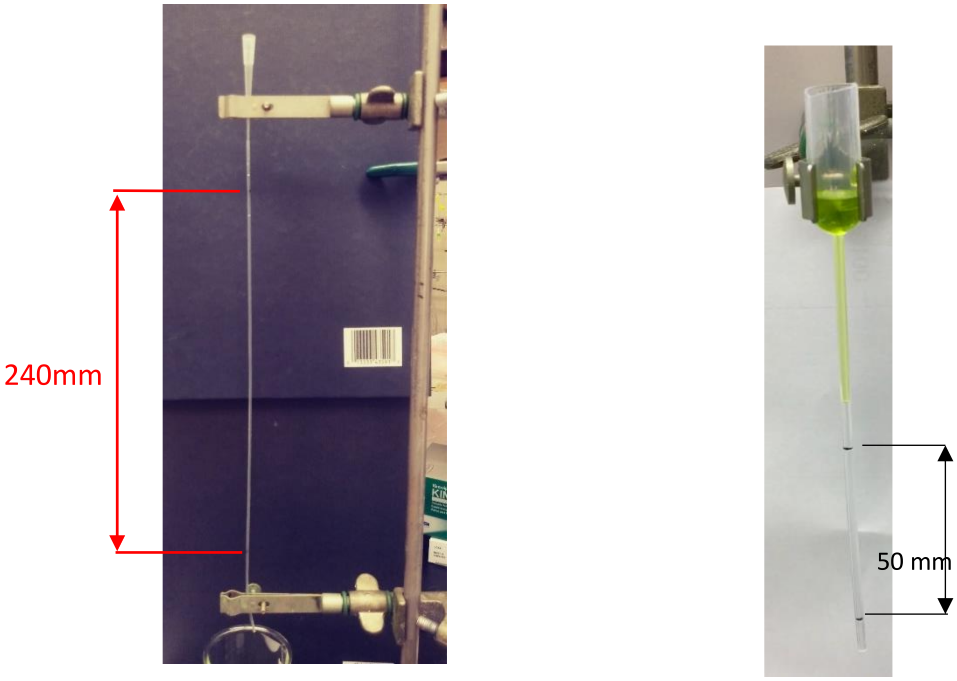

Figure S1: Home-built, disposable viscometers. Setup A, used for low viscosity samples, consisted of tubing with an inner diameter of 1.19 mm and timing marks separated by 240 mm. Setup B, used for high viscosity samples, consisted of a wide bore pipette (Fisher cat # 13-711-6M), with the top removed for easy loading, and timing marks separated by 50 mm.

Standardized Curves

A B

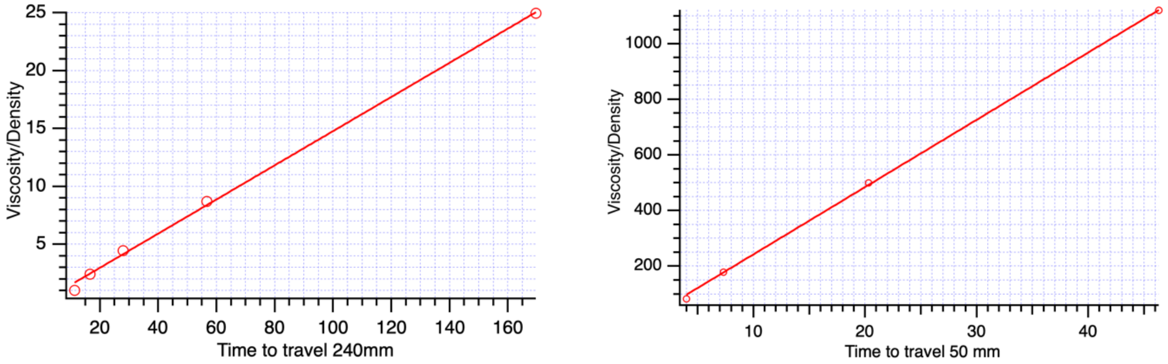

Figure S2: Standard curves for viscosity determination. Standardized solutions of glycerol and water were loaded onto a (**A**) long, narrow tube and (**B**) wide bore pipette. Markings on each set were spaced 240mm apart on the long tube and 50mm apart on the pipette. The time was measured for the liquid standards to travel between the markings.

RAW DATA

Supplemental Table 1: Raw data of saliva samples. The time required for samples to travel 240 mm (**A**) or 50 mm (**B**) measured in seconds. The processed samples (**A**) were loaded onto viscometer Setup A, while the unprocessed (**B**) were loaded onto viscometer Setup B.

A

**Homogenized saliva samples**	**112.76**	**242.91**	**194.4**
	19	14	15
	20	13	15
	20	13	15

B

**Unprocessed Saliva samples**	**112.76**	**242.91**	**194.4**
	8	27	7
	8	28	7
	8	26	7

Supplemental Table 2: Viscosity of saliva samples. The viscosity of the saliva samples in centipoise (cP) were determined from the average travel time of the samples between viscometer timing marks (as measured in Suppl Table 1), sample density, and comparison with the standardized curve of the particular viscometer (Suppl Fig 1).

Setup A

**Processed Sample**	**Average time for 240mm (s)**	**Density (g/mL)**	**Viscosity (cP)**
**112.76**	19.67	1.067	3.10
**242.91**	13.33	1.053	2.07
**194.4**	15	1.112	2.46

Setup B

**Unprocessed Sample**	**Average time for 50mm (s)**	**Density (g/mL)**	**Viscosity (cP)**
**112.76**	8	1.014	196.23
**242.91**	26	1.077	677.37
**194.4**	7	1.045	176.95

Weight Distribution

Supplemental Table 3: Weight Distribution. Unprocessed samples were weighed on an analytical balance (mg) to determine the total weight. Material with a low enough viscosity to enter a plastic pipette was weighed (Measured viscous material). The weight of the higher viscosity material was calculated as the difference between the weight of measured viscous material from the total weight. The Percent of useable saliva was calculated by dividing measured viscous material by total weight.

Unprocessed sample	112.76	242.91	194.4
Total weight	422.96	719.41	1021.57
Measured viscous material	416.31	586.01	625.54
High viscous material	6.65	133.4	396.03
Percent of useable saliva	98.4	81.5	61.2

.
